# Molecular Implications of the PPARs in the Diabetic Eye

**DOI:** 10.1155/2013/686525

**Published:** 2013-02-04

**Authors:** Andreea Ciudin, Cristina Hernández, Rafael Simó

**Affiliations:** Unidad de Investigación en Diabetes y Metabolism, CIBER de Diabetes y Enfermedades Metabólicas Asociadas (CIBERDEM), Instituto de Salud Carlos III, Instituto de Investigación Vall d'Hebron, Universitat Autónoma de Barcelona, Paseo Vall d'Hebron, 119-129 Barcelona, Spain

## Abstract

Diabetic retinopathy (DR) remains as the leading cause of blindness among working age individuals in developed countries. Current treatments for DR (laser photocoagulation, intravitreal corticosteroids, intravitreal anti-VEGF agents, and vitreoretinal surgery) are applicable only at advanced stages of the disease and are associated with significant adverse effects. Therefore, new pharmacological treatments for the early stages of the disease are needed. Emerging evidence indicates that peroxisome proliferator-activator receptors (PPARs) agonists (in particular PPAR**α**) are useful for the treatment of DR. However, the underlying molecular mechanisms are far from being elucidated. This paper mainly focuses on PPARs expression in the diabetic eye, its molecular implications, and the effect of PPAR agonists as a new approach for the treatment of DR. The availability of this new strategy will not only be beneficial in treating DR but may also result in a shift towards treating earlier stages of diabetic retinopathy, thus easing the burden of this devastating disease (Cheung et al. (2010)).

## 1. Introduction

Diabetic retinopathy is the most common complication of diabetes, and proliferative diabetic retinopathy (PDR) remains the leading cause of blindness among working-age individuals in developed countries [1]. Diabetic macular edema (DME), another important event that occurs in diabetic retinopathy, is more frequent in type 2 than type 1 diabetes [2]. Although PDR is the most common sight-threatening lesion in type 1 diabetes, DME is the primary cause of poor visual acuity in type 2 diabetes. Because of the high prevalence of type 2 diabetes, DME is the main cause of visual impairment for diabetic patients. In addition, DME is almost invariably present when PDR is detected in type 2 diabetes [3]. Despite heterogeneity in patient selection criteria, country and selection period, the prevalence of patients with DR in Western countries is relatively similar, ranging from 21.9 to 36.8% [4]. Population-based studies suggest that about one-third of the diabetic population have signs of DR and one-tenth have vision-threatening states of retinopathy such as diabetic maculae edema (DME) and proliferative diabetic retinopathy (PDR) [5, 6]. 

Neovascularization caused by severe hypoxia is the hallmark of PDR, whereas vascular leakage caused by the breakdown of the blood retinal barrier (BRB) is the main event involved in the pathogenesis of DME.

Healthcare costs for patients with DR are almost double than that of patients without it and they increase considerably with the severity of DR [7, 8], which suggests that preventing the progression of DR may significantly reduce the economic burden related to this complication of diabetes [9].

Current treatments for DR (laser photocoagulation, intravitreal corticosteroids, intravitreal anti-VEGF agents, and vitreoretinal surgery) are applicable only at advanced stages of the disease and are associated with significant adverse effects [10, 11]. Therefore, new pharmacological treatments for the early stages of the disease are needed. 

In recent years, several experimental and clinical studies have shown the beneficial effects of peroxisome proliferator-activator receptors (PPARs) agonists (in particular PPAR*α*) in diabetic retinopathy. However, the molecular mechanisms are far from being elucidated. In this paper, we review PPARs expression in the diabetic eye, its molecular implications, and the effect of PPAR agonists as a new approach for the treatment of DR.

## 2. Peroxisome Proliferator-Activator Receptors (PPARs) and Their Agonists: A General Overview

Peroxisome proliferator-activator receptors (PPARs) are members of the nuclear hormone receptor superfamily of ligand-activated transcription factors that regulate gene expression in response to nutritional and physiological stimuli. 

The nuclear receptor superfamily can be divided into two categories: first, the classic hormone receptors that bind specific hormones (glucocorticoids, thyroid hormones, and estrogen) and second, nuclear receptors that act as metabolic sensors, binding to substrate or end-products of metabolic pathways, such as the liver X receptors, the farnesoid X receptor, hepatocyte nuclear factor 4*α*, or PPARs [12].

PPARs were initially identified as a peroxisome proliferator “binding-protein” capable of inducing hepatocyte peroxisome proliferation. Peroxisomes are subcellular organelles whose main function is the removal of molecular oxygen and breaking down hydrogen peroxide [13, 14]. However, the peroxisomes are also involved in glycerolipid synthesis, fatty-acid oxidation, glucose and cholesterol biosynthesis and metabolism [15]. 

The PPARs function together with the retinoid X receptor (RXR) is to regulate glucose and lipid metabolism. In the presence of the specific ligands, the PPARs adopt an active conformation by forming a heterodimer with the RXR, resulting in binding to peroxisome proliferator response elements in target genes, determining PPAR-dependent gene expression [16]. A PPAR is a compact molecule, consisting of 5 or 6 structural regions (A–F) divided into four functional domains. The C-domain of the PPARs is DNA-binding and the E or F region is the ligand-binding site. The ligand-binding site has a pivotal role in transcriptional activation [15].

Despite the high levels of homologies at the protein level, three isoforms have been identified, each of which has different numbers of amin oacid residues: PAAR*α* (468 amino acids residues), PAAR *β*/*δ* (441 amino acids), and PPAR*γ* (479 amino acids) [17]. They are distributed differently in the body tissues and exert different functions. 

PPAR*α* is highly expressed in tissues with elevated mitochondrial and peroxisomal fatty-acid beta-oxidation rates, such as liver, heart muscle, kidney, skeletal muscle, brown fat, and retina [18–20]. PPAR*α* is also present in monocytes, macrophages, and endothelial cells [21]. 

PPAR *β*/*δ* are distributed ubiquitously in almost all tissues and recent data suggests their involvement in cell proliferation, angiogenesis, and inflammation [22]. PPAR*γ* has been intensively studied for its crucial implication in glucose homeostasis and insulin sensitivity [23]. PPAR*γ* is also involved in the regulation of lipid metabolism by increasing the genes that regulate fatty-acid uptake and storage [24] and plays a pivotal role in adipocyte differentiation and function [25].

So far seven PPAR*γ* isoforms have been identified, most of them (PPAR*γ*1, PPAR*γ*2, PPAR*γ*3, PPAR*γ*6, and PPAR*γ*7) expressed abundantly in adipose tissue. PPAR*γ*4 and PPAR*γ*5 are expressed only in macrophages [23]. 

### 2.1. PPAR*α* Agonists

The first PPAR discovered was PPAR*α*, during the search of a molecular target for a group of agents then referred to as *peroxisome proliferators*, as they increased peroxisomal numbers in rodent liver tissue, apart from improving insulin sensitivity. These receptors, pharmacologically related to the fibrates, were discovered in the early 1980s [26]. The main natural ligands of PPAR*α* are the fatty acids and endogenous eicosanoids. Physiological concentrations of diet-derived unsaturated fatty acids also activate PPAR*α* [19]. In addition, PPAR*α* are strongly stimulated by synthetic molecules such as the fibrates, a class of amphipathic carboxylic acids (gemfibrozile, clofibrate and fenofibrate). The main effects of PPAR*α*-stimulation in humans are decreasing triglycerides, shifting low-density lipoprotein cholesterol to larger particles, and increasing high-density lipoprotein cholesterol particles.

### 2.2. PPAR*γ* Agonists

The main natural ligands of PPAR*γ* are the fatty acids, the phospholipids and their oxidatively modified metabolites, and a group of natural nitroalkenes [27]. One of the natural ligands of PPAR*γ* is an oxidatively modified phospholipid, 15-deoxy-*δ* 12,14-prostaglandin J2 (15d-PGJ2) and there are also other J2 series prostaglandins. In general, the endogenous ligands have a relatively low affinity and show little specificity towards the different PPARs. Additionally, most of the effects of PGJ2 occur independent of PPAR*γ*.

 In 1995, a class of antidiabetic drugs, the thiazolidinediones (TZDs), were shown to activate PPAR*γ* with high affinity, even more effectively than any natural ligand [28]. Various synthetic ligands for PPAR*γ* were developed, such as the TZD family: ciglitazone, troglitazone, rosiglitazone, and pioglitazone. Some non-TZD synthetic ligands such as GW1929 and GW7845 are mainly used in experimental research. 

### 2.3. Dual *α*/*γ* Agonists

In order to combine the beneficial effects of both PPAR*α* and PPAR*γ* activation, dual *α*/*γ* agonists have recently been developed. Synthetic molecules, like muraglitazar or tesaglitazar, were shown to be superior to TZDs in terms of improving glucose metabolism and raising HDL-c levels in T2D patients, but the phase III clinical trials have been discontinued [29]. A meta-analysis of the phase II and III clinical trials of muraglitazar revealed that it was associated with a greater incidence of myocardial infarction, stroke, transient ischemic attacks, and CHF when compared to placebo or pioglitazone [30]. Tesaglitazar was generally well tolerated but was associated with a greater increase in serum creatinine level than placebo [31]. At present, aleglitazar, a dual PPAR *α*/*γ* agonist, currently in phase III clinical development, seems to be a potent and balanced activator of PPAR*α* and *γ*, with beneficial effects in type 2 diabetic patients who have suffered a recent cardiovascular event [32, 33].

 Telmisartan, an angiotensin-II receptor (AT-1) blocker, has been proven to be a dual PPAR *δ*/*γ* agonist, and bezafibrate is a pan-PPAR agonist. Furthermore, telmisartan was shown to have a beneficial effect in a murine model of retinal neovascularization, by promoting a dual AT-1 blockade and PPAR*γ* activation, suggesting a possible implication in the treatment of DR [34, 35]. 

Several angiotensin-II receptor blockers (candesartan, irbesartan, losartan, but not valsartan or olmesartan) were shown to activate PPAR*γ* ligands *in vitro*. However, only telmisartan, and to a lesser extent candesartan, resulted in significant PPAR*γ* agonism in cell cultures, but *in vivo*, sartan treatment had no effect as insulin sensitizers [36] suggesting a very weak PPAR*γ* stimulation *in vivo*.

### 2.4. Dual *α*/*δ* Agonists

Molecules targeting both PPAR*α* and PPAR*δ* were also developed. GFT505 is a dual *α*/*δ* agonist currently completing a phase II clinical trial. It shows a good tolerance and a significant improvement of lipid and glucose disorders associated with metabolic syndrome [37]. 

## 3. Distribution of the PPARs in the Retina

All of the PPARs are constitutively expressed in the whole retina [38] but most of our knowledge refers to the retinal pigment epithelia (RPE). It has recently been shown that PPARs are expressed in cultures of primary RPE cells and ARPE19 cells (a human immortalized line of RPE cells) [39]. Both cell types presented a lack of PPAR*γ*2 and moderate PPAR*γ*1 and PPAR *β*/*δ* expression. The ARPE-19 cells showed a moderate expression of PPAR*α* while primary RPE cells had a low representation. Interestingly, the same study analyzed the PPARs in the freshly isolated RPE, and the results were slightly different from the studies of cell cultures: a lack of PPAR*γ*1 and PPAR*γ*2 expression and a high representation of PPAR*α* and PPAR *β*/*δ*. However, the results of the freshly isolated RPE might have been altered due to the small number and the significant heterogeneity of the sample and are in contrast to the findings of previous studies which demonstrated the presence of PPAR*γ* in the RPE [40]. These conflicting results suggest that further studies addressed to evaluating the distribution of the PPARs not only in RPE, but also in the neuroretina are needed. 

## 4. Molecular Implications of PPAR*γ* Activation in Diabetic Retinopathy 

PPAR*γ* activation plays a key role in the development of DR. In fact, several experimental studies have shown that PPAR*γ* receptors are downregulated in the diabetic eye and that their suppression is involved in the pathogenesis of DR [41, 42]. In addition, there is clinical evidence to suggest that some PPAR*γ* polymorphisms represent a genetic risk factor for developing DR [43, 44]. 

PPAR*γ* activation might participate in abrogating the two most important events that occur in DR: neurodegeneration and microangioapathy. 

### 4.1. Effect of PPAR*γ* Activation in Retinal Neurodegeneration

Although DR has been classically considered to be a microcirculatory disease of the retina, there is emerging evidence to suggest that retinal neurodegeneration is an early event in the pathogenesis of DR which antedates and participates in the microcirculatory abnormalities that occur in DR [45, 46]. In this regard, it is worth mentioning that the main features of retinal neurodegeneration (apoptosis and glial activation) have been found in the retinas of diabetic donors without any microcirculatory abnormalities appearing in the ophthalmoscopic examinations performed during the year before death [47, 48]. Retinal ganglions cells (RGCs), located in the inner retina, are the retinal neurons in which the apoptosis process related to diabetes is first detected [49]. Regarding glial activation, it is important to note that a complex milieu of dysregulated proinflammatory factors occurs in the diabetic retina, and while retinal microglia and infiltrating monocytic cells probably make an important contribution, there is also strong evidence that Müller glia shows inflammation-linked responses when exposed to the diabetic milieu [50, 51]. 

Glutamate, the major excitatory neurotransmitter in the retina is an essential mediator of the retinal neurodegenerative process that occurs in DR. Glutamate has been found elevated in the extracellular space in experimental models of diabetes [52–55]. This extracellular and synaptic excess of glutamate leads to the overactivation of the ionotropic glutamate receptors, mainly alpha-amino-3-hydroxyl-5-methyl-4-isoxazole-propionate (AMPA) and N-methyl-D-aspartame (NMDA) receptors, which results in an uncontrolled intracellular calcium response in postsynaptic neurons and cell death [56, 57]. This deleterious effect of glutamate on retinal neurons is known as “excitotoxicity” and retinal ganglion cells are primarily affected. 

Aoun et al. [58] demonstrated that two PPAR*γ* ligands, 15d-PGJ2 and troglitazone protect RGC-5 (an established transformed rat retinal ganglion cell line) against glutamate insult. The neuroprotective effects of the two compounds appeared to be mediated through an antioxidant rather than a PPAR-gamma-dependent pathway. In addition, PPAR*γ* is constitutively expressed in rat primary microglial cultures and PPAR*γ* activation had an anti-inflammatory effect [59, 60]. Furthermore, it has recently been demonstrated that rosiglitazone attenuates diabetes-induced apoptosis in retinal neurons of STZ-induced diabetic rats by inhibition of phospho-STAT3 (p-STAT3) and cytokine signaling 3 (SOCS3) [61].

Apart from the neuroprotection due to the abrogation of excitotoxicity, there is emerging evidence that PPAR*γ* activation also inhibits the neurotoxicity resulting from the overexpression of the renin-angiotensin system that exists in DR [62–65].

Finally, activation of PPARgamma may play an important role in regulating the expression of target genes that are involved in lipid and fatty acid metabolism in the photoreceptor renewal process. The RPE is a specialized epithelium lying in the interface between the neural retina and the choriocapillaris where it forms the outer blood-retinal barrier (BRB). However, the RPE is something more than a constituent of the BRB and the phagocytosis of shed photoreceptor membranes is vitally important for maintaining the structural and functional integrity of the retina [66]. In fact, photoreceptor homeostasis is maintained mainly by the RPE's phagocytic capacity, which is able to degrade between 25.000 and 30.000 outer segment disks daily [67]. The RPE plays a central role in the processing and recycling of fatty acids and other lipids resulting from photoreceptor digestion. Ershov et al., using primary cultures from rat RPE cells, demonstrated that photoreceptor phagocytosis selectively activates PPAR*γ* expression while having no effect on PPAR*α* or PPAR *β*/*δ* [67]. This result suggests the implication of PPAR*γ* in preventing the possible deleterious effect of fatty acid accumulation in the retina, thus conferring neuroprotection.

### 4.2. Effect of PPAR*γ* Activation on Microangiopathy

Apart from neuroprotection, PPAR*γ* activation has beneficial effects on early microvascular abnormalities. The loss of pericytes and endothelial dysfunction are the hallmarks of these early stages of diabetic microangiopathy, and the balance between nitric oxide (NO) [vasodilator] and endothelin-1 (ET-1) [vasoconstrictor] is essential in determining the hemodynamic response of the capillaries. One of the most important functional impairments in the early stages of DR is the reduced production and bioavailability of NO [68] and the increase of ET-1 [69]. A study focused on NO production in pericytes showed that PPAR*γ* is constitutively expressed in the retinal pericytes and that troglitazone increased NO production in a PPAR*γ*-dependent manner [70]. In addition, it has been shown that pioglitazone elicits the endothelium-dependent dilation of isolated porcine retinal arterioles mediated by NO release [71]. Furthermore, PPAR*γ* activation inhibits ET-1 secretion from endothelial cells [72]. Taken together, PPAR*γ* activation exerts a clear beneficial effect on the imbalance between NO and ET-1 that exists in DR.

VEGF plays an essential role in the pathogenesis of DR by leading to the disruption of the BRB (the main pathogenic event in the development of DME) and by promoting neovascularisation (the hallmark of PDR) [73]. Therefore, the effect of PPAR*γ* agonists on VEGF plays a key role in accounting for the clinical and experimental results of PPAR*γ* agonists, in particular TZDs in the development of both DME and PDR.

The relationship between TZDs and DME has generated an intense debate that is still far from being elucidated. Clinical evidence indicates that TZDs significantly increase the risk of heart failure by a PPAR*γ*-mediated mechanism which leads to fluid retention. Because of this effect, there has been concern about the possible relation between the use of TZDs and DME. There are several studies which support this theory, especially in patients having impaired renal function, cardiac failure, or associated insulin therapy [74–78]. Fong and Contreras [77] in a large prospective cohort study showed that TZDs users were more likely to develop DME (OR, 2.6 [95% CI, 2.4–3.0]). Idris et al. [78], in a retrospective cohort study of 103,368 patients with type 2 diabetes mellitus and without DME at baseline, showed at 1 year, an incidence of DME of 1.3% (*n* = 41) and 0.2% (*n* = 227) among thiazolidinedione users (*n* = 3227) and nonusers (*n* = 100,141), respectively. After Cox multiple regression analysis, multiple imputation analysis to adjust for missing values, and propensity score analysis to exclude any selection bias, TZD use was associated with an increased risk of DME at 1-year followup (OR, 2.3 [95% CI, 1.5–3.6]) and 10-year followup (HR, 2.3 [95% CI, 1.7–3.0]). The effect was similar for pioglitazone and rosiglitazone. Combination therapy with insulin plus TZD was associated with a higher risk of DME after propensity score adjustment (HR, 3.0 [95% CI, 1.5–5.9]). By contrast in the ACCORD-eye substudy, no association was observed between TZD exposure and DME in patients with type 2 diabetes [79]. In this study, the cross-sectional association of DME and visual acuity with TZD was examined by means of baseline fundus photographs and visual acuity measurements from the ACCORD trial. TZD use was not associated with DME in unadjusted (odds ratio [OR], 1.01; 95% confidence interval [CI], 0.71–1.44; *P* = .95) and adjusted (OR, 0.97; 95% CI, 0.67–1.40; *P* = .86) analyses. In addition, TZD use was associated with slightly greater visual acuity (0.79 letter; 95% CI, 0.20–1.38; *P* = .009) but this effect was of uncertain clinical significance. Nevertheless, it is worthy of mention that the ACCORD-EYE study had an important selection bias because patients at potential risk for macular edema were excluded [80]. There is little experimental information on the relationship between PPAR*γ* activation and DME development. Muranaka et al. [81] demonstrated that rosiglitazone was effective in protecting against the breakdown of the BRB in streptozotocin-induced diabetic rats but this effect was not mediated by VEGF downregulation. By contrast, Zheng at al. [82] have recently found that simvastatin decreases retinal vascular permeability in streptozotocin-induced diabetic rats through the inhibition of VEGF expression and p38MPAK activity mediated by the PGC-1*α*. It was demonstrated that PPAR*γ* activation by PIO upregulates PGC-1*α* suggesting a protective effect of [83] PPAR*γ* activation on the breakdown of the BRB induced by diabetes. Taken together, current evidence regarding the relationship between PPAR*γ* activation and BRB function is confusing and further clinical and experimental studies addressed to examining this issue are urgently required. 

The role of PPAR*γ* activation in VEGF-induced PDR is also worthy of discussion. Several studies showed a proangiogenic effect of PPAR*γ* activation by the increased expression of VEGF, suggesting a possible deleterious effect in PDR [84–86]. However, these studies were performed in human vascular muscle cell [84], adipocytes [85], and cultured cardiac myofibroblasts [86]. The heterogeneity of the behaviour of endothelial cells in several body compartments is well known. Therefore, the results obtained from a certain cell type cannot be extrapolated to another. In the retina, it seems that PPAR*γ* activation has an antiangiogenic effect. Murata et al. [87] using the oxygen-induced ischemia (OII) mouse model of retinal neovascularization showed that PPAR*γ* activation by intravitreous administration of troglitazone or rosiglitazone inhibited VEGF-mediated neovascularisation. Interestingly, VEGF was not significantly inhibited in the ganglion cell layer, thus preserving the neuroprotective properties of VEGF in this critical neural layer. Aljada et al. [88] demonstrated by using chick chorioallantoic membrane (CAM) model that rosiglitazone and pioglitazone inhibited the proangiogenic effects of bFGF (basic fibroblast growth factor) and VEGF. Higuchi et al. [89] found that pioglitazone attenuated pathological retinal microvessel formation in a mouse model of OII through adiponectin-mediated modulation of TNF*α* production. More recently, Rodrigues et al. [90] using ARPE-19 cell cultures demonstrated that PPAR*γ* agonists can have differential effects on RPE survival in response to oxidative stress: troglitazone but not rosiglitazone or pioglitazone was able to improve the RPE response to oxidative stress by downregulating VEGF expression. Finally, Hatanaka et al. [91], have shown that pioglitazone inhibits fibrotic change in primary monkey RPECs through the suppression of TGF-*β* signaling. A clinical study which supports these experimental findings was conducted by Shen et al., [92] who showed in a case-control study that rosiglitazone reduced the progression from NPDR to PDR over 3 years by 59%. 

Apart from the potential beneficial effects on neurodegeneration and microvascular abnormalities, PPAR*γ* activation might also counteract other mechanisms involved in the pathogenesis of DR such as inflammation and leukostasis [81, 93, 94], the overexpression of matrix metalloproteinases [95] or the increase in platelet aggregation [96]. 

## 5. Molecular Implications of the PPAR*α* in Diabetic Retinopathy 

Ever since 1969 there has been clinical evidence of a beneficial effect of PPAR*α* activation on diabetic retinopathy. Harrold et al. showed an improvement in retinal exudate after 1 year of treatment with clofibrate, without significant effects on other retinal lesions [97]. The data was confirmed by Dorne in 1977, who suggested clofibrate as the treatment of choice for exudative diabetic retinopathy [98]. 

The current evidence that PPAR*α* activation has a beneficial effect in DR comes from two seminal clinical trials: the FIELD [99, 100] and the ACCORD-Eye [101] studies which showed that DR progression was significantly reduced by fenofibrate (a PPAR*α* used as a hypolipemiant agent). 

The FIELD Study raised the prospect of the prevention of DR via treatment with fenofibrate. FIELD was essentially a cardiovascular trial, with a large population (*n* = 9795) of type 2 diabetes patients without statin treatment at baseline randomised to receive fenofibrate or placebo for 5 years [99]. Primary and secondary endpoints focussed on cardiovascular events. Eight per cent of the population of FIELD had retinopathy at baseline, and the need for laser photocoagulation for retinopathy was included among the *a priori *tertiary endpoints of the trial [99]. Fenofibrate reduced the incidence of patients requiring laser photocoagulation (from 5.2% on placebo to 3.6%, *P* = 0.0003). There was greater absolute benefit in patients with, rather than without, pre-existing retinopathy [100]. However, baseline photographic assessment of retinal status was only made in 10% of patients. Moreover, the criteria for the use of laser treatment were not pre-specified and therefore are likely to have been heterogeneous [102].

A substudy conducted in 1012 patients explored the effects on retinopathy outcomes in the FIELD study in more detail [100]. In this ophthalmological substudy, retinopathy status and severity were assessed from two-field 45° colour fundus photographs of the macula (stereoscopic) and a disc/nasal field taken at baseline, 2 years and 5 years, and graded with Early Treatment Diabetic Retinopathy Study (ETDRS) criteria. A marked and significant reduction (~70%) in the risk of laser treatment for retinopathy was again demonstrated for fenofibrate versus placebo. However, only 28 patients required laser treatment (23 in the placebo group and 5 in the fenofibrate group). In addition, DR progression, (2-step Early Treatment Diabetic Retinopathy Study [ETDRS] scale, the primary endpoint), was significantly reduced with fenofibrate in those patients with preexisting DR at baseline (from 14.6% to 3.1%, *P* = 0.004), but not in those without DR at baseline. However, the number of events was small (14 in the placebo group and 3 in the fenofibrate group). 

The ACCORD trial included a lipid arm, in which patients were randomly assigned to treatment with fenofibrate or placebo in addition to open-label simvastatin [103]. Patients eligible for this arm were also enrolled in the glycemia evaluation, but met additional recruitment criteria relating to lipids (LDL cholesterol 1.55–4.65 mmol/L, HDL cholesterol <1.29 mmol/L [<1.42 mmol/L for women], and triglycerides <8.5 mmol/L [<4.5 mmol/L if receiving lipid-modifying therapy]). Retinopathy outcomes in ACCORD were evaluated in a 4-year eye substudy [101]. Randomization to fenofibrate relative to placebo (on background therapy with simvastatin) was associated with a significant decrease (from 10.2% to 6.5%, *P* = 0.006) in DR progression (3 or more steps on the EDTRS), with greater effect in patients with evidence of DR at baseline (absolute RR 6.9% versus 0.2% in those without DR at baseline). It should be noted that the reduction obtained with fenofibrate plus simvastatin (−40%; *P* = 0.006) was even higher than that obtained in the arm of intensive glycaemic control (−33%; *P* = 0.003).

The similarities between the effects of fenofibrate on outcomes in the eye between the FIELD and ACCORD-EYE studies are striking. In both trials, randomization to fenofibrate (in combination with a statin in a substantial proportion of patients by study end in FIELD, and exclusively with a statin in ACCORD-EYE) led to statistically and clinically significant reductions in the risk of a range of clinical endpoints related to retinopathy [99–101]. In summary, these trial data show that fenofibrate treatment provides a relative reduction in DR progression of 30–40% over 4 to 6 years, with greater benefit in patients with pre-existing DR. These benefits were achieved despite a lack of significant reductions in the risk of the primary composite cardiovascular endpoint in either study (although there was a significant reduction in total cardiovascular events in FIELD) [99, 103].

### 5.1. Mechanisms of Action of Fenofibrate

#### 5.1.1. Lipid-Mediated Mechanisms

Fenofibrate is indicated for the treatment of hypertriglyceridemia and mixed dislipidemia. Its main action is to lower plasma triglyceride levels, but it also reduces total and LDL cholesterol, raises HDL cholesterol, and decreases the concentration of small LDL cholesterol particles and apolipoprotein B. In the FIELD study, there was essentially no change in HDL-cholesterol with fenofibrate (mean reduction of 0.01 mmol/L for fenofibrate versus placebo at study end) and only a modest change in triglycerides (mean reduction 0.24 mmol/L) [99]. In ACCORD, mean HDL-cholesterol increased from 0.98 mmol/L to 1.07 mmol/L with fenofibrate and from 0.99 mmol/L to 1.05 mmol/L with placebo [103]. Similarly, baseline and final triglyceride levels were 1.85 mmol/L and 1.38 mmol/L in the fenofibrate group of the ACCORD lipid arm, compared with 1.81 mmol/L and 1.63 mmol/L in the placebo group. Therefore, it seems that the beneficial effects of fenofibrate on diabetic retinopathy are unrelated to quantitative changes of serum lipids. However, it is not known whether the effectiveness of fenofibrate in modulating the qualitative properties of lipoproteins (i.e., reducing remnants and small dense LDL particles) can contribute to its beneficial effects. 

It should also be noted that the mechanisms regulating intraretinal lipid transport, rather than serum lipid levels, might be more important in the pathogenesis of DR [102]. In this regard, we have recently shown that apolipoprotein A1 (apo-A1) is overexpressed in the retina of diabetic patients [104, 105]. Apo-A1 is a key factor for the intraretinal transport of lipids, thus preventing lipid deposition and lipotoxicity, and is also a potent scavenger of reactive oxygen species. Therefore, apo-A1 could play an important role in protecting the retina from oxidative stress. These findings have led us to hypothesize that the retinas of diabetic patients have a higher content of apo-A1 as a protective mechanism and, consequently, that those patients with less capacity for apo-A1 production by the retina will be more prone to develop lipid deposition (hard exudates) and retinal damage induced by oxidative stress. Fenofibric acid has been shown to enhance transcription of the apoA-1 gene in the liver [106], macrophages, and fibroblasts [107], but whether this is also true at the retinal level remains to be elucidated.

Finally, it has recently been shown that circulating apoAI may be an independent protective factor for the development of DR [108]. Therefore it is possible that the increase in apoAI plasma levels induced by fenofibrate participates in its beneficial action on DR.

#### 5.1.2. Nonlipidic-Mediated Mechanisms

There are several nonlipidic mechanisms by which fenofibrate, or its active metabolite, fenofibric acid (FA) can exert beneficial effects in preventing or arresting DR. 

### 5.2. Neuroprotective Effect

As previously mentioned, neurodegeneration plays an essential role in the pathogenesis of DR. In experimental models of cerebral ischaemia and neurodegenerative diseases, PPAR*α* activation had a neuroprotective effect, independent of lipid metabolism [109]. Antioxidant, anti-inflammatory, and antiapoptotic properties of fenofibrate have been implicated in this effect. 

### 5.3. Improvement in Endothelial Function and Anti-Apoptotic Activity

FA exerts a protective effect on the microvasculature by suppressing apoptosis and stimulating nitric oxide synthase (eNOS) phosphorylation and NO production. This is mediated by AMPK activation, as has been shown by studies of numerous cell systems, including human retinal endothelial cells [110–113]. This effect is unrelated to PPAR*α* activation, as is evident in human retinal endothelial cells [113]. In addition, a recent study showed that FA elicits dual protective effects in the RPE by the down-regulation of stress-mediated signalling and the induction of autophagy and survival pathways [114]. 

### 5.4. Antioxidant and Anti-Inflammatory Activity

Fenofibrate may mitigate the adverse effects of oxidative and inflammatory stress, which are involved in the development of DR. It has been reported that PPAR*α* activation induces the expression and activation of antioxidant enzymes, such as superoxide dismutase and glutathione peroxidase [109], thus ameliorating oxidative stress, a key factor for the development of DR [115]. 

PPAR*α* activation induces the apoptosis of human monocyte-derived macrophages [116] and inhibits the expression of vascular cell adhesion molecules on the endothelium [117]. These effects might be potentially relevant in preventing leukostasis. Furthermore, it has recently been shown that FA prevents the deleterious action of IL-1*β* in the disruption of the BRB, thus supporting the role of proinflammatory cytokines in the pathogenesis of DME [118].

### 5.5. Preventive Effects on Blood-Retinal Barrier Breakdown

The breakdown of the BRB, due to the disruption of tight junctions with subsequent leakage, is the main factor implicated in DME. Fenofibrate prevents DME progression, suggesting a possible effect in reducing the permeability associated with DR. In cultures of human RPE cells (which constitute the external BRB), FA prevented the disorganisation of tight junction proteins and hyperpermeability provoked by the diabetic milieu. This is mediated by the effect of FA in reducing interleukin-*β*-induced AMPK activation [118]. Finally, it has recently been shown that FA down-regulates the overexpression of basement membrane components (fibronectin and collagen IV) in RPE cells cultured in conditions mimicking the diabetic milieu. Exposure to FA reduced the increase in permeability associated with the overexpression of fibronectin and collagen IV in a dose-dependent manner [119]. 

### 5.6. Antiangiogenic Activity

PPAR-*α* is present in endothelial cells [120], and its activation by means of PPAR-*α* agonists has recently been shown to inhibit expression of VEGF receptor 2 (VEGFR2) and neovascularization in human umbilical endothelial cells [121]. Varet et al. [122] have demonstrated that fenofibrate inhibits angiogenesis *in vitro* and *in vivo* as well as basic fibroblast growth factor-induced angiogenesis *in vivo*. In addition, in cells derived from human ovarian cancer, clofibric acid (a PPAR-*α* agonist) downregulates VEGF expression [123]. Finally. Chen et al. [124] have recently shown that both oral and intravitreal administration of fenofibrate ameliorated leukostasis and retinal vascular leakage in type 1 murine models, and that they also attenuated the overexpression of adhesion molecules and VEGF. The beneficial effects of fenofibrate were blocked by a specific PPAR*α* antagonist, thus suggesting a PPAR*α*-dependent mechanism.

## 6. Molecular Implications of the PPAR *β*/*δ* in Diabetic Retinopathy

PPAR *β*/*δ* is also expressed in the retina. Little is known about the effects of PPAR *β*/*δ* in the eye and less about its effects in the diabetic eye. Most of the studies have associated PPAR *β*/*δ* activation with proangiogenic and proinflammatory effects. The PPAR *β*/*δ* agonist GW501516 stimulates human umbilical vein endothelial cells proliferation and increased VEGF expression [125].

## 7. Conclusions

Improvements in diabetes care and management have been crucial in lowering the incidence and severity of DR. Nevertheless, DR remains the most common cause of vision impairment in working age adults in the US and Europe and retinal neovascularization occurs in up to 20% of patients with diabetes. As greater knowledge of the molecular mechanisms involved in the pathogenesis of DR has been obtained, new therapeutic products have been developed. In this regard, scientific evidence has accumulated in recent years regarding the role of PPAR activation in the pathogenesis of DR. As far as we know, PPAR*γ* activation would appear to have a beneficial effect in the early stages of DR. The beneficial effects of PPAR*α* activation by fenofibrate in DR have been demonstrated in two large clinical trials (FIELD and ACCORD-EYE) with greater benefit in patients with pre-existing DR. By contrast, PPAR *β*/*δ* activation has a deleterious effect, promoting inflammation and angiogenesis. However, the underlying mechanisms by which PPARs are exerting their effects in the retina are only just beginning to be understood. 

Concerted efforts to better define the presence and distribution of PPARs in freshly isolated human retina and specific studies addressed to examining the mechanistic pathways and functional effects involved in PPAR activation in both nondiabetic and diabetic retina are urgently needed. 

Finally, improved understanding of the mechanisms of action of PPARs will facilitate their clinical application. Ophthalmologists and physicians treating diabetic patients should be aware of the potential usefulness of PPARs and work together not only in future research, but also in establishing clinical guidelines that will include these drugs as medical treatments for DR. Only such coordinated action, together with rational strategies targeting prevention, will be effective in reducing the burden of DR and improving clinical outcomes related to this devastating complication of diabetes.
